# Mindfulness-Based Cognitive Therapy (MBCT) Reduces the Association Between Depressive Symptoms and Suicidal Cognitions in Patients With a History of Suicidal Depression

**DOI:** 10.1037/ccp0000027

**Published:** 2015-08-24

**Authors:** Thorsten Barnhofer, Catherine Crane, Kate Brennan, Danielle S. Duggan, Rebecca S. Crane, Catrin Eames, Sholto Radford, Sarah Silverton, Melanie J. V. Fennell, J. Mark G. Williams

**Affiliations:** 1 Department of Psychiatry, Warneford Hospital, University of Oxford; 2 Centre for Mindfulness Research and Practice, Bangor University; 3 Department of Psychiatry, Warneford Hospital, University of Oxford

**Keywords:** suicidality, depression, mindfulness, recurrence, cognitive reactivity

## Abstract

***Objective:*** In patients with a history of suicidal depression, recurrence of depressive symptoms can easily reactivate suicidal thinking. In this study, we investigated whether training in mindfulness, which is aimed at helping patients “decenter” from negative thinking, could help weaken the link between depressive symptoms and suicidal cognitions. ***Method:*** Analyses were based on data from a recent randomized controlled trial, in which previously suicidal patients were allocated to mindfulness-based cognitive therapy (MBCT), an active control treatment, cognitive psychoeducation (CPE), which did not include any meditation practice, or treatment as usual (TAU). After the end of the treatment phase, we compared the associations between depressive symptoms, as assessed through self-reports on the Beck Depression Inventory–II (Beck, Steer, & Brown, 1996), and suicidal thinking, as assessed through the Suicidal Cognitions Scale (Rudd et al., 2001). ***Results:*** In patients with minimal to moderate symptoms at the time of assessment, comparisons of the correlations between depressive symptoms and suicidal cognitions showed significant differences between the groups. Although suicidal cognitions were significantly related to levels of symptoms in the 2 control groups, there was no such relation in the MBCT group. ***Conclusion:*** The findings suggest that, in patients with a history of suicidal depression, training in mindfulness can help to weaken the association between depressive symptoms and suicidal thinking, and thus reduce an important vulnerability for relapse to suicidal depression.

## Abstract (public health significance statement toc summary)

This study suggests that mindfulness-based cognitive therapy can help to uncouple the link between suicidal thinking and depressive symptoms in patients who have suffered from suicidal depression in the past. This is important, as a strong coupling between depressive symptoms and suicidal cognitions has been suggested to increase risk for relapse to suicidal depression. It remains unclear from the current study, however, how stable these changes are and how far they translate into actual reductions in rates of relapse.

Suicidality often recurs with depressive episodes (Ten Have et al., 2009). In fact, of all the noncore symptoms of depression (symptoms other than negative mood and anhedonia), suicidality has been found to be the one that reemerges most consistently: If patients have been suicidal during a given episode of depression, they are very likely to become suicidal again the next time they are depressed (Williams, Crane, Barnhofer, Van der Does, & Segal, 2006). Even if not resulting in suicidal behavior, suicidality represents a serious outcome in its own right. Recurrence of suicidal ideation has wider, worrisome implications, as the risk for detrimental outcomes increases with the number of previous episodes of suicidality (Van Orden et al., 2010). It is of paramount importance, therefore, to find interventions that can impede escalation of depressive symptoms into suicidality. Mindfulness-based cognitive therapy (MBCT; Segal, Williams, & Teasdale, 2002) has been shown to be effective in reducing risk for relapse and recurrence in depression (Piet & Hougaard, 2011). Here we were interested more specifically in investigating whether the treatment can help to reduce vulnerability for relapse to suicidal depression.

In individuals who have been depressed in the past, maladaptive patterns of thinking can easily become reactivated through minor triggers, such as subtle changes in mood (Segal et al., 2006; Segal, Gemar, & Williams, 1999; Scher, Ingram, & Segal, 2005). It has been suggested that the propensity for this cognitive reactivity develops as a consequence of associative learning during previous episodes of depression (Segal, Williams, Teasdale, & Gemar, 1996). As depressed patients repeatedly engage in negative thinking, associations between negative mood and the patterns of thinking that are prevalent in this mood are formed and strengthened. The particular content and pattern that get associated with negative mood depend on what is part of the “rehearsal pool.” Following initial suggestions by Beck (1996), Rudd (2000 and 2006) has introduced the concept of a suicidal mode, referring to a cognitive–affective–behavioral network that, once activated, guides synchronous processes that manifest on a number of different levels, including suicidal cognitions, negative affect, physiological arousal, and motivation to engage in suicidal behavior. In line with this view, research has shown that, under conditions of low mood, previously depressed patients with a history of suicidality are significantly more likely to show suicidal thinking and related cognitive deficits than previously depressed patients without such a history (Williams, Barnhofer, Crane, & Beck, 2005; Williams, Van der Does, Barnhofer, Crane, & Segal, 2008). According to Rudd (2000), the cognitive component of the suicidal mode is characterized by three categories of core beliefs: unlovability, helplessness, and poor distress tolerance. Activation of these beliefs, and ensuing perceptions of helplessness, inadequacy, and inability to cope are assumed to increase risk for suicidal ideation and behavior. Recent research has demonstrated that cognitions related to these three categories of core beliefs significantly predict suicidal ideation over and above depressive symptoms and general levels of hopelessness (Ellis & Rufino, 2015; Rudd et al., in press).

MBCT has been specifically designed to help participants become better able to recognize and disengage from maladaptive patterns of thinking. Training in mindfulness aims to serve this purpose by cultivating a metacognitive mode that allows patients to “decenter” from negative thinking, that is, to observe their “thoughts and feelings as temporary, objective events in the mind, as opposed to reflections of the self that are necessarily true” (Fresco et al., 2007). Consistent with this rationale, research into the mechanisms of MBCT for the prevention of depression relapse has demonstrated that increases in the ability to decenter significantly mediate effects of the treatment on rates of relapse (Bieling et al., 2012). The aim of the current analyses was to investigate whether mindfulness training can provide an effective tool for weakening the link between depressive symptoms and suicidal cognitions related to the core beliefs of the suicidal mode described by Rudd (2000).

To test this hypothesis, we analyzed data from a randomized, controlled trial of MBCT for relapse prevention that included a large number of participants with a history of suicidal depression. Participants in this trial had to have a history of at least three previous episodes of major depression. As risk of relapse is known to increase with number of previous episodes (Mueller et al., 1999; Solomon et al., 2000), use of this criterion ensured that participants were likely to be at high risk for relapse. It has been suggested that cognitive vulnerability processes become increasingly easier to trigger with repeated recurrence of depression (Segal et al., 1996), and that to prevent relapse in individuals with a history of repeated episodes, mental training is of particular importance. Previous trials of MBCT have found that the training has significant preventive effects in patients with three or more previous episodes, but there is little evidence for beneficial effects in patients with two or fewer previous episodes (Piet & Hougaard, 2011). The Staying Well After Depression Trial (Williams et al., 2010) compared the effects of MBCT to two control conditions: an active control treatment, cognitive psychoeducation (CPE), in which participants received all of the elements of MBCT except the meditation practice, and treatment as usual (TAU). Patients reported levels of suicidal cognitions related to the core beliefs of unlovability, helplessness, and poor distress tolerance as measured on the Suicidal Cognitions Scale by Rudd, Joiner and Rajab (2001), assessed before and after the treatment phase. This provided the opportunity to investigate effects on the association between current depressive symptoms and suicidal cognitions. We hypothesized that following MBCT, suicidal reactivity, as indicated by correlations between symptoms of depression and suicidal cognitions, would be significantly lower than following treatment with CPE (which included verbal *discussion* of decentering but no mindfulness *practice*) and TAU, which was unlikely to include any systematic training of or reference to decentering strategies.

## Method

The study protocol for the trial was approved by the Oxfordshire Research Ethics Committee C. All participants provided written consent before any research activity.

### Participants and Study Flow

We assessed eligibility using the *Structured Clinical Interview for* DSM–IV: *Research Version* (*SCID*; First, Spitzer, Gibbon, & Williams, 2002), which was conducted by formally trained research assistants. Inclusion criteria for the trial were (a) age between 18 and 70 years, (b) a history of at least three previous episodes of depression, meeting *Diagnostic and Statistical Manual of Mental Disorders* (4th ed., text rev*.*) criteria (*DSM–IV*-TR; American Psychiatric Association, 2000), two of which must have occurred within the last 5 years and one within the last 2 years, and (c) being in remission during the previous 8 weeks. If potential trial participants reported that at least 1 week during the previous 8 they had experienced either a core symptom of depression (depressed mood, anhedonia) or suicidal feelings, plus exhibited at least one other symptom of depression, they were deemed *not* to be in recovery or remission, and hence ineligible for the study. For the current analysis, which focused on recurrences of suicidal thinking, we included only those participants who had reported a previous history of suicidal ideation or suicidal behavior as assessed by the *SCID*.

Exclusion criteria were (a) history of schizophrenia, schizoaffective disorder, bipolar disorder, current abuse of alcohol or other substances, organic mental disorder, pervasive developmental disorder, or regular nonsuicidal self-injury; (b) positive continuing response to cognitive–behavioral therapy (CBT), due to the known effects of CBT in reducing risk of relapse (Hollon, Stewart, & Strunk, 2006), which could have potentially confounded treatment effects in the trial; (c) current psychotherapy or counseling more than once a month; (d) regular meditation practice (meditating more than once per month); or (e) inability to complete research assessments because of difficulty with English, visual impairment, or cognition. As part of the trial, interviewer reliability for *SCID* diagnoses (First et al., 2002) of depression was assessed by independent ratings of a sample of 91 follow-up interviews conducted by two independent psychiatrists, which yielded an agreement of κ = .74, 95% CI [0.60, 0.87] between the original assessor and the independent rater.

Participants provided information on all measures described in this study at baseline (pretreatment), following which they were randomly assigned to one of the three treatment arms: MBCT, CPE or TAU. Patients in MBCT and CPE received an individual preclass interview and eight weekly 2-hr sessions; patients in TAU received an initial interview session, in which the importance of treatment seeking was stressed. Most baseline assessments were scheduled within a 3-month window between treatment cohorts prior to the beginning of the interventions (*M* = 58.79, *SD* = 31.38 days); most posttreatment assessments were scheduled within a 6-week window following the end of the interventions (*M* = 29.52, *SD* = 19.53). We followed the standard protocol for MBCT (Segal et al., 2002) except that we placed greater emphasis on patterns and thoughts associated with suicidality, its escalation, and preparation of an action plan to respond to suicidal crisis. CPE comprised all elements of the MBCT program except for the experiential cultivation of mindfulness through meditation practice. Details of the trial protocol are given in Williams et al. (2010); the main results of the two-center trial are reported in Williams et al. (2014).

### Outcome Measures

Severity of depressive symptoms was measured using the Beck Depression Inventory-II (BDI-II; Beck, Steer, & Brown, 1996). Current levels of suicidal cognitions were measured using the Suicidal Cognitions Scale (SCS; Rudd et al., 2001).

#### Beck Depression Inventory–II

The BDI-II (Beck et al., 1996) consists of 21 groups of statements, referring to the presence of symptoms of depression over the past 2 weeks. Internal consistency in the current sample was α = .88 at pretreatment, and .90 at posttreatment.

#### Suicidal Cognitions Scale

The SCS (Rudd et al., 2001) consists of questions referring to core beliefs reflecting the suicidal mode of thinking described by Rudd (2000). Factor analyses conducted by Rudd et al. (in press) have indicated factors related to the suicidal schemas of unbearability (e.g., “I can’t stand this pain anymore”) and unlovability (e.g., “I am completely unworthy of love”). Recent studies by other authors (Bryan et al., 2014; Ellis & Rufino, 2015) suggest a third factor reflecting unsolvability (“Suicide is the only way to end this pain”), which is in line with the initial theoretical suggestions by Rudd (2000). Items of the self-report questionnaire are rated on a Likert-type scale scored from 1 to 5. In the current study we used the 20-item version of the scale that was published by Rudd et al. (2001). This version differs from a recently revised version of the questionnaire, in which Rudd et al. (in press) removed items containing the word “suicide” (i.e., Items 2 and 15, which both load highly on the unsolvability factor identified by Ellis & Rufino, 2015, and Bryan et al., 2014) to reduce construct overlap for studies predicting suicidal ideation and behavior. Results in our study remained unchanged when analyses were based on this revised version. All analyses focused on the total scale. Internal consistency of this scale in the current sample was α = .93 at pretreatment, and .94 at posttreatment, which is comparable to findings from previous studies (Ellis & Rufino, 2015; Rudd et al., in press). Ellis and Rufino (2014) reported a test–retest reliability of *r* = .46, *p* < .01, over the course of treatment in a sample of psychiatric inpatients. In the current sample, test–retest reliability from pre- to posttreatment was *r*(192) = .50, *p* < .001.

## Results

### Demographic and Clinical Characteristics

Information on patient demographics and clinical characteristics is presented in Table 1. Of the 274 individuals who participated in the trial, 221 reported past suicidality (ideation or behavior), 194 of whom had provided full data sets at both pre- and postassessment. Seventy-seven of these participants had been assigned to MBCT, 78 to CPE, and 39 to TAU. There were no significant differences between the three treatment groups in terms of sociodemographic variables or course characteristics (see Table 1, all *p* > .05). At entry into the study, patients in this trial subsample had a mean age of 43.7 years (*SD* = 12.1). Women comprised 74%, and 95% were of Caucasian ethnicity. Clinical course of the disorder was characterized by a mean age of onset of 20.1 (*SD* = 10.1). Participants had suffered from an average of 7.3 (*SD* = 5.5) previous episodes of depression. Those who had dropped out of treatment showed no significant differences in sociodemographic variables and course characteristics from those who had provided data at posttreatment (all *p* > .10).[Table-anchor tbl1]

### Scatterplots and Frequency Distributions

**Figure 1 fig1:**
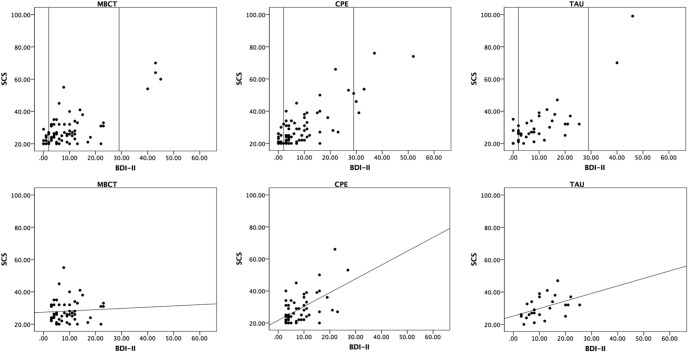
Scatterplot and regression lines of Beck Depression Inventory-II (BDI-II) (Beck et al., 1996) and Suicidal Cognitions Scale (SCS) scores (Rudd et al., 2001) by group at posttreatment. The top panel shows scores of all participants with past suicidality (MBCT: *n* = 77, CPE: *n* = 78, TAU: *n* = 39). The bottom panel shows scores and regression lines in groups of participants with BDI-II ≥ 3 and ≤ 28 (MBCT: *n* = 52, CPE: *n* = 53, TAU: *n* = 26). MBCT = mindfulness-based cognitive therapy; CPE = cognitive psychoeducation; TAU = treatment as usual.

Inspection of the scatterplots of the relation between BDI-II (Beck et al., 1996) and SCS (Rudd et al., 2001) scores at posttreatment (see Figure 1, upper panel) showed a number of cases with extreme values that would have disproportionately determined linear relations. We decided to deal with these outliers by excluding participants with severe levels of symptoms, using the conventional upper boundary for moderate symptoms on the BDI-II, a score of 29, as a cutoff. This is in keeping with the theoretical focus of the study on suicidal reactivity, or more specifically on the question of whether treatment can reduce the association between depressive symptoms and suicidal cognitions that becomes obvious already with presence of minimal to moderate symptoms of depression. There were four participants in the MBCT group, five in the CPE group, and two in TAU with scores above 29, indicating comparable rates of outlying across the groups, 6%, 6%, and 5%, respectively. All of these participants showed high levels of suicidal cognition, suggesting that, in these cases, the treatments had neither prevented relapse into depression nor into suicidality. Participants with severe symptoms at posttreatment showed significantly higher numbers of previous episodes, *F*(1, 165) = 4.85, *p* = .02, η^2^ = .02 (*M* = 11.22, *SD* = 11.30, in participants with BDI-II scores above 29, *M* = 7.10, *SD* = 4.97, in participants with BDI-II scores of 29 or lower), but did not differ with regard to all other sociodemographic and course characteristics (all *p* < .10). [Fig-anchor fig1]

Inspection of the scatterplots and frequency distributions also showed that there was a considerable number of participants in each of the groups who showed no or extremely low levels of symptoms. As activation of suicidal cognitions is unlikely to occur when patients show few or no symptoms, inclusion of these participants could have artificially inflated linear relations between symptoms and suicidal cognitions. For this reason, we decided to run analyses including only participants with a BDI-II score of 3 or higher, indicating presence of at least one symptom at full expression over the past 2 weeks. To explore the robustness of findings, we then reran analyses including participants with scores of at least 2, 1, and, eventually, 0. In the MBCT group, there were 21 participants (27%) with a BDI-II score of 2 or less, 17 participants (22%) with a score of 1 or less, and 10 (13%) with a score of 0. In the CPE group, there were 19 participants (24%) with a score of 2 or less, 17 participants (21%) with a score of 1 or less, and 12 (15%) with a score of 0. In the TAU group, 13 participants (33%) had a score of 2 or less, 11 (28%) had a score of 1 or less, and 5 (12%) had a score of 0. Participants with a score of less than 3 on the BDI-II did not differ significantly in sociodemographic and course characteristics from those with BDI-II scores of 3 or more (all *p* < .10, apart from a trend for age of onset, *F*(1, 192) = 3.12, *p* = .07, η^2^ = .01; participants with BDI-II scores of less than 3: *M* = 17.96 (*SD* = 7.86), participants with BDI-II of 3 or more: *M* = 20.87 (*SD* = 10.78). The lower panel of Figure 1 depicts the scatterplot of the relation between BDI-II (Beck et al., 1996) and SCS scores (Rudd et al., 2001) for the more restricted sample of participants who reported minimal to moderate symptoms of depression, that is, BDI-II scores ≥ 3 and ≤ 29.

### Changes in BDI and SCS Scores

Means and standard deviations of BDI-II (Beck et al., 1996) and SCS scores (Rudd et al., 2001) at pre- and postassessment in participants with minimal to moderate symptoms are listed in Table 2. A 2 (time) × 3 (group) repeated-measures ANOVA of BDI-II scores showed no significant effects for group, time or their interaction (all *p* > .10). An identical analysis for SCS scores showed a significant main effect for group, *F*(1, 129) = 16.88, *p* = .00, η^2^ = .11, which was qualified by a significant Group × Time interaction, *F*(2, 129) = 4.2, *p* = .02, η^2^ = .06, due to significant reductions in the MBCT group, mean pre- to postdifferences (*M*_(I-J)_) = 8.53, *SE* = 1.61, *p* = .00; there were no significant differences in the CPE group, *M*_(I-J)_ = 2.93, *SE* = 1.58, *p* = .07, or the TAU group, *M*_(I-J)_ = 1.74, *SE* = 2.28, *p* = .44. These results remained substantially unchanged when analyses were conducted with changes in BDI-II scores entered as a covariate, thus suggesting that changes in SCS scores were not entirely due to reductions in depressive symptoms. When the above analyses were conducted using BDI-II lower cut-offs of 2 and 1, respectively, follow-up analyses also showed significant reductions in SCS scores in the CPE group. When analyses were conducted including participants with a score of 0, the Time × Treatment interaction for SCS scores reached only trend levels, *F*(2, 179) = 2.83, *p* = .06, η^2^ = .03, due to relatively more pronounced reductions in the two control groups, *M*_(I-J)_ = 2.98, *SE* = 1.76, *p* = .09, in the TAU group, and, *M*_(I-J)_ = 3.15, *SE* = 1.26, *p* = .01, in the CPE group, compared with *M*_(I-J)_ = 6.95, *SE* = 1.25, *p* = .00, in the MBCT group. [Table-anchor tbl2]

Changes in SCS (Rudd et al., 2001) scores in the MBCT group showed a marginally significant correlation with the amount of meditation practice during the time of the intervention (mean time of daily formal meditation practice reported by the participants), *r*(50) = –.26, *p* = .05.

### Investigation of Group Differences in the Relation Between BDI-II and SCS Scores

To investigate the relation between posttreatment BDI-II (Beck et al., 1996) and SCS scores (Rudd et al., 2001) within the three groups, we computed zero-order correlations. There were significant associations in the CPE group, *r*(52) = .60, *p* = .00, and in the TAU group, *r*(24) = .45, *p* = .02, but no significant association in the MBCT group, *r*(50) = .06, *p* = .67. Fisher *r*-to-*z* transformations indicated significant differences between correlations in the MBCT and the CPE group, *z* = 3.19, *p* = .000, and the MBCT and the TAU group, *z* = 1.67, *p* = .04; there was no significant difference between correlations in the CPE and TAU groups, *z* = .86, *p* = .19.

When we computed correlations in groups defined by lower boundaries for BDI-II inclusion scores, we found slightly higher correlations in the MBCT group *r*(54) = .11 for BDI-II ≥ 2, *r*(61) = .18 for BDI-II ≥ 1, and *r*(71) = .30 for BDI ≥ 0, while correlations in the CPE group, *r*(54) = .60 for BDI-II ≥ 2, *r*(59) = .61 for BDI-II ≥ 1, and *r*(71) = .65 for BDI-II ≥ 0, and the TAU group, *r*(35) = .50 for BDI-II ≥ 2, *r*(30) = .50 for BDI-II ≥ 1, and *r*(24) = .52 for BDI-II ≥ 0, remained at the same level. The pattern of differences remained largely unchanged, except that *r*-to-*z* transformations reflecting differences between correlations in the MBCT group and the TAU group reached only trend levels in groups with a lower boundary of 1, *z* = −1.62, *p* = .05, or 0, *z* = −1.32, *p* = .09.

The fact that the difference between correlations in the MBCT group and the TAU group was less robust may, at least partly, have been due to the smaller size of the TAU group resulting from the recruitment ratio of 2:2:1. As we did not assume significant differences in the relation between BDI-II (Beck et al., 1996) and SCS scores (Rudd et al., 2001) in the CPE and in the TAU group, we reran analyses comparing the MBCT group with both of the control groups together. This yielded a correlation coefficient of *r*(78) = .56, *p* = .00, in the control groups, which was significantly different from the coefficient of *r*(50) = .06, *p* = .67, found in the MBCT group, *z* = −3.13, *p* = .00. The difference between correlations in the two groups remained significant when tested across all the different lower boundaries for BDI-II inclusion scores, MBCT: *r*(54) = .11, control groups: *r*(86) = .56, *z* = −2.98, *p* = .00, for BDI ≥ 2; MBCT: *r*(61) = .18, control groups: *r*(91) = .57, *z* = −2.78, *p* = .00, for BDI ≥ 1; MBCT: *r*(71) = .30, control groups: *r*(108) = .61, *z* = −2.60, *p* = .00, for BDI ≥ 0.

## Discussion

It has been suggested that, in patients with a history of suicidal depression, changes in mood can reactivate a suicidal mode of processing, reflected in cognitions revolving around core beliefs of unlovability, helplessness, and poor distress tolerance. To investigate whether training in mindfulness can uncouple this link, in the current study, we investigated the relations between levels of depressive symptoms and levels of suicidal cognitions in different treatment groups of a randomized, controlled trial. In line with our hypothesis, there was a significantly weaker association between levels of symptoms and suicidal cognitions in the group who had received MBCT than in the groups who had received treatments that did not include training in mindfulness. Furthermore, despite the fact that levels of depressive symptoms remained relatively unchanged, participants in the MBCT group showed a general reduction in suicidal cognitions, suggesting that in participants of this group, suicidal cognitions were less likely to spiral in response to the occurrence of depressive symptoms.

Interpretation of these findings requires taking into account a number of limitations. Of note, the study assessed the association of depressive symptoms and suicidal cognitions at a single predetermined point in time, which may or may not have represented the most relevant period with regard to risk for recurrence. Indeed variation at this point was such that we had to exclude a considerable number of participants who showed no or only very low levels of symptoms and would thus have been very unlikely to experience activation of a suicidal mode. Because the distribution of levels of symptoms in remitted samples tends to be skewed, with lower numbers of individuals showing relatively higher levels of depression, relatively high sample sizes are needed to demonstrate robust relations, which is likely to account for our observation that the difference between the MBCT and the CPE group was more robust than the difference between the MBCT and the TAU group (recruited to be only half the size of the other two groups).

Furthermore, analyses excluded participants who showed severe symptoms. Although there was no reason to assume that cases with severe symptoms did not represent true observations, inspection of the scatterplots within the MBCT group identified these cases as outliers that would have disproportionately determined linear relations. Exclusion of these cases is in line with the focus of the study on vulnerability to relapse and suicidal reactivity as compared with the question of whether relapse to depression continues to be associated with suicidality. As the current analyses focused on effects at the end of treatment, it remains unclear to which degree the relapse of these participants was due to adverse effects or ineffectiveness of the treatment, or the fact that presence of severe symptoms might have precluded effective participation in the training.

It is also important to note that the current study was based on a sample of participants who had been in full remission at entry into the trial. Many patients with a recurrent course of the disorder show residual symptoms between episodes (Judd et al., 1998; Paykel et al., 1995) and it is possible that this criterion might have excluded those who are most reactive. This is likely to have worked against our chances of finding strong effects. In fact, previous research has found that MBCT produced better effects in patients with unstable remissions as compared with those with stable remissions (Segal et al., 2010).

Because of the use of a dismantling design, in which MBCT was compared with a control treatment, CPE, that included all the elements of MBCT except the mindfulness training, it is possible to attribute the observed differences in association between MBCT and CPE as specifically due to the effects of mindfulness practice. Reactivation of suicidal cognitions can occur relatively automatically, and this finding supports the assumption that systematic mental training is needed to help patients to become better at decentering from the habitual maladaptive patterns of thinking that arise as a consequence of cognitive reactivity.

Increasing the ability to decenter is one of the main aims of MBCT and the current findings suggest that participants can learn to effectively transfer this skill to respond to suicidal cognitions. This is important, as past research on cognitive interventions has shown that positive effects in alleviating depression do not necessarily transfer to reduce vulnerability for suicidality (Cuijpers et al., 2013). We find it interesting that the version of MBCT used in the current trial did not include any major modifications to specifically address suicidality. Instead, the training followed the general principle of working with the material that occurs spontaneously during meditations, assuming that spontaneous occurrence of difficulties provides the most important indicator of their relevance. Because of this, the extent to which participants had opportunities for learning and practicing new responses to suicidality may have differed widely between participants and therapy groups. It is possible, therefore, that inclusion of exercises that address more specifically and explicitly the problem of suicidal reactivity might further increase the potential of MBCT to reduce vulnerability for suicidal depression. Although decreases in coupling between depressive symptoms and suicidal cognitions should reduce risk of relapse to suicidal depression, it was not possible to test relations between reactivity and relapse in the current data, as variation in symptoms between participants precluded inferences about individual differences in reactivity. Comparisons between groups showed no significant differences in rates of relapse to suicidal depression at the end of the follow-up of the trial in patients who had reported a history of suicidality: MBCT, *n* = 12 (13%); CPE, *n* = 18 (20%); TAU, *n* = 11 (25%); χ^2^ (2, 221) = 2.78, *p* = .24).

With regard to the scope of the current findings, it is important to keep in mind that the current study was an investigation of reactivation of suicidal cognitions in response to minimal to moderate levels of depression, and in a group of participants with a history of suicidality that, in most cases, was characterized by suicidal ideation, but neither behavior nor attempts. Mindfulness may also be helpful in the context of more significant levels of symptoms and in patients with more severe histories of suicidality, but such contexts often require significantly different approaches to those used in the current context. For example, dialectical behavior therapy, which was developed to treat people with borderline personality disorder and chronic suicidality (Linehan, Armstrong, Suarez, Allmon, & Heard, 1991), offers mindfulness training as an adjunct to a broad range of other interventions aimed at dealing with emotional instability, destructive impulsivity, and chaotic life problems.

Altogether, the current results suggest that, within the context of relapse prevention, mindfulness training can effectively uncouple the link between depressive symptoms and suicidal cognitions in individuals who are at risk for suicidality, and thus reduce an important vulnerability for relapse to suicidal depression. Further research will have to investigate how stable the described effect is, and how far it translates into actual reductions in rates of relapse.

## Figures and Tables

**Table 1 tbl1:** Sociodemographic Characteristics

Variable	MBCT (*n* = 77)	CPE (*n* = 78)	TAU (*n* = 39)
Female, *n* (%)	57 (74)	60 (76)	28 (71)
Age at enrolment, *M (SD)*	44.5 (11.8)	43.1 (12.2)	43.2 (12.5)
Ethnicity (Caucasian), *n* (%)	74 (96)	72 (92)	39 (100)
Employed at enrollment, *n* (%)	52 (67)	45 (57)	26 (66)
Age of first onset of major depression, *M (SD)*	19.3 (8.5)	20.2 (10.6)	21.4 (12.6)
Number of previous episodes, *M (SD)*	8.0 (7.4)	7.0 (3.2)	6.4 (4.7)
Antidepressant use at enrollment, *n* (*%*)	31 (40)	33 (42)	14 (35)
SCID current or past alcohol abuse/substance abuse/dependence, *n* (%)	13 (16)	12 (15)	8 (20)
SCID current or past anxiety disorder, *n* (%)	28 (36)	31 (39)	15 (38)
*Note*. MBCT = mindfulness-based cognitive therapy; CPE = cognitive psychoeducation; TAU = treatment as usual. SCID = *Structured Clinical Interview for* DSM-IV *Axis I*.			

**Table 2 tbl2:** Means and Standard Deviations of BDI and SCS Scores at Pre- and Posttreatment

Measures and groups	Pretreatment	Posttreatment
BDI, *M (SD)*		
MBCT (*n* = 52)	9.2 (7.4)	9.0 (5.4)
CPE (*n* = 54)	10.6 (10.0)	9.0 (6.5)
TAU (*n* = 26)	9.0 (6.8)	11.4 (6.3)
SCS, *M (SD)*		
MBCT (*n* = 52)	36.6 (14.3)	28.1 (7.0)^a^
CPE (*n* = 54)	32.4 (9.1)	29.5 (9.7)
TAU (*n* = 26)	32.2 (9.4)	30.4 (6.6)
^a^ Simple main effects with pretreatment score *p* < .001, all other simple main effects *p* > .05. BDI = Beck Depression Inventory; SCS = Suicidal Cognitions Scale; MBCT = mindfulness-based cognitive therapy; CPE = cognitive psychoeducation; TAU = treatment as usual.
